# Simultaneous occurrence of acute posterior shoulder dislocation and posterior shoulder-fracture dislocation after epileptic seizure

**DOI:** 10.4103/0973-6042.57937

**Published:** 2009

**Authors:** Cem Copuroglu, Bilal Aykac, Beyti Tuncer, Mert Ozcan, Erol Yalniz

**Affiliations:** Trakya University, Medical Faculty, Department of Orthopaedics and Traumatology, Edirne, Turkey

Sir,

Complex posterior shoulder-fracture dislocation is a rare injury.[1,2] Epileptic seizures can cause shoulder dislocation and glenohumeral instability, but a characteristic pattern of instability and structural lesion is not well known. Glenohumeral instability following seizures is usually of the posterior type.[3] Bilateral fractures of the proximal humerus are rare, and extremely rare in combination with posterior dislocation of the shoulder joint. Impression fractures of the humeral articular surface are common with this injury.[4] The humeral head becomes wedged behind the glenoid, and its anteromedial aspect is eroded (reverse Hill Sachs lesion). Posterior dislocation of the shoulder may be associated with an anteromedial impaction fracture rendering the shoulder unstable in internal rotation. Shoulder-fracture dislocation is often associated with poor long-term function regardless of the choice of treatment.[1]

A 69-year-old, right hand–dominant man presented to our clinic with bilateral ecchimosis and disability of shoulder movements. As reported by him, a day before, he had sustained an injury to both his shoulders while he was sleeping. There was no history of trauma, and he did not have any knowledge about his epileptic seizures. As his wife said, he had some convulsions while he was sleeping; and after he woke up, he had difficulty in moving his arms and pain around his shoulders.

**Figure 1:**
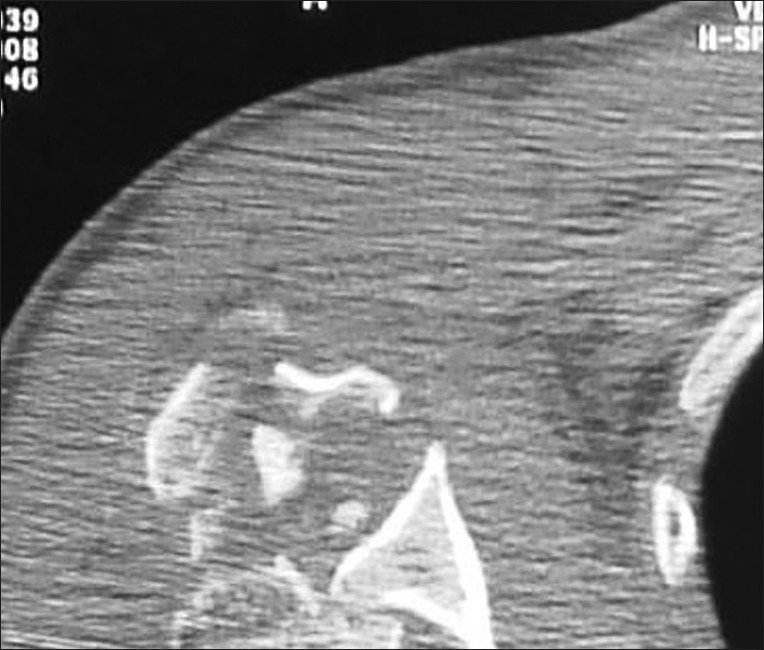
Computerized tomography scan of right shoulder

**Figure 2:**
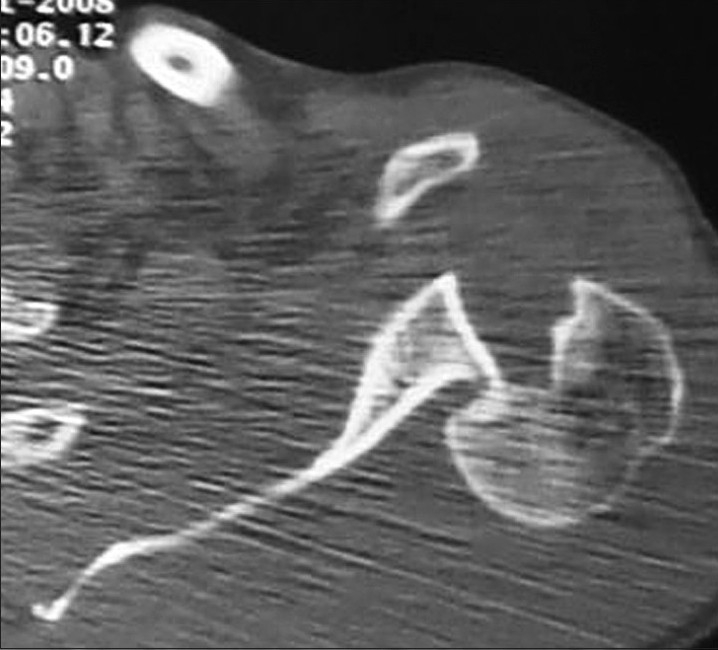
Computerized tomography scan of left shoulder

Physical examination revealed a large ecchimosis around both shoulders; left shoulder was in internal rotation position, he had pain and limitation of motion and right shoulder had severe pain, crepitation and limitations of movements in all directions. The patient had painful restriction of both active and passive glenohumeral movements in all directions. No neurovascular impairment was found. Plain radiographs showed posterior shoulder dislocation with a bone defect of the anteromedial humeral head on the left side and multiple fragmented proximal humerus fracture with posterior dislocation on the right side. Computed tomography showed many pieces of proximal humerus with posterior dislocation of the right shoulder [Figure 1] and locked posterior dislocation with reverse Hill Sachs lesion (approximately 30% of the humeral head) of the left shoulder [Figure 2].

**Figure 3:**
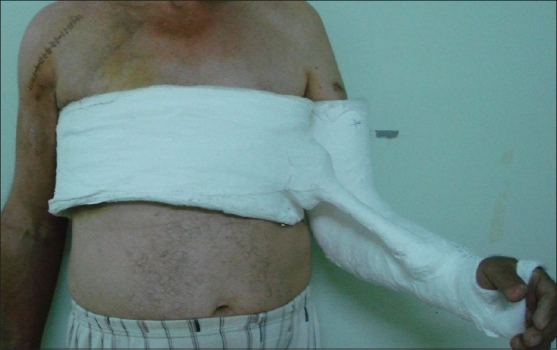
Right shoulder hemiarthroplasty, left shoulder in a cast in external rotation

**Figure 4:**
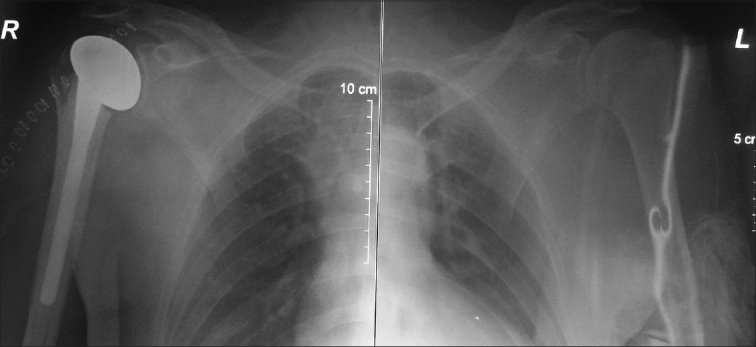
Postoperative roentgenograms of both shoulders

Both his arms were stabilized in arm slings, and surgical intervention was planned. On the third day, under general anesthesia, the patient was placed in supine position and the left shoulder was reduced with closed manipulation with the help of the image intensifier. It was seen that it was stable during slight external rotation. A cast with his arm in minimal abduction and almost 15 degrees of external rotation was applied [Figure 3]. For the right shoulder, in the semi-fowler (beach chair) position, partial shoulder hemi-arthroplasty was applied [Figure 4].

Postoperatively, passive shoulder exercises were begun immediately for the right shoulder, and the left shoulder was immobilized in external rotation for 4 weeks. After the cast was removed, the left shoulder was stable in internal and external rotation. The result of the apprehension test was negative. He could abduct his right arm 80 degrees and left arm 100 degrees.

Four months after the operation, the right shoulder abduction was 60 degrees, forward flexion was 60 degrees and extension was 20 degrees without pain. Left shoulder abduction was 160 degrees; forward flexion160 degrees; extension, 30 degrees; and internal rotation, 55 degrees without instability. His epilepsy was managed by the Neurology Department.

Posterior shoulder dislocations and posterior shoulder-fracture dislocations are uncommon injuries that most often occur during seizures or as a result of high-energy trauma.[1] In the absence of trauma, posterior shoulder-fracture dislocation (unilateral or bilateral) is virtually pathognomonic of a seizure. Treatment consists of closed relocation of the shoulder followed by assessment of its stability. Stability is assessed by internal rotation of the shoulder to establish the point at which redislocation occurs. The degree of instability is usually determined by the size of the anterior humeral head defect, as it reengages with the posterior aspect of the glenoid rim, although instability can also be produced by posterior capsular injury. If the reduction is stable throughout a functional range of rotation (usually in a patient with a dislocation accompanied by a small humeral head defect occupying <25% of the articular surface), immobilization of the shoulder in neutral or external rotation for 4 weeks is recommended.[1] The shoulder is in its most stable position when it is in neutral forward elevation and external rotation. This is the generally preferred position of immobilization following relocation of the shoulder to prevent acute redislocation.

If the shoulder redislocates as it is internally rotated, operative stabilization is recommended because of the shortcomings of immobilization, which include shoulder stiffness, acute redislocation and recurrent instability.[1] Furthermore, splinting of the arm in external rotation is difficult to maintain, unless a permanent shoulder spica is applied.

The treatment is determined by the age and medical status of the patient and the degree of devascularization and fragmentation of the humeral head and tuberosities. It has been suggested that for defects that involve less than 20% of the articular surface, closed reduction can be attempted. Open reduction is necessary for defects that involve 20% to 40% of the surface. Hemi-arthroplasty or total shoulder replacement is generally regarded as a better option for fracture dislocations and for multipart fractures in the elderly, as they offer rapid recovery.

In this case, we applied hemi-arthroplasty for the fracture dislocation side, and we followed the locked posterior dislocation with a reverse Hill Sachs lesion (defect, almost 30%) side with nonoperative treatment in a cast in external rotation. The functional outcome was good. Posterior shoulder dislocations with large humeral head defects can be treated with closed reduction and immobilized in the stable position for 4 weeks. We think that conservative treatment should always be attempted before surgical intervention.
